# Sex-specific molecular differences in glioblastoma: assessing the clinical significance of genetic variants

**DOI:** 10.3389/fonc.2023.1340386

**Published:** 2024-01-23

**Authors:** Nicolina Jovanovich, Ahmed Habib, Akanksha Chilukuri, N. U. Farrukh Hameed, Hansen Deng, Regan Shanahan, Jeffrey R. Head, Pascal O. Zinn

**Affiliations:** ^1^ Hillman Cancer Center, University of Pittsburgh Medical Center, Pittsburgh, PA, United States; ^2^ Department of Neurosurgery, University of Pittsburgh Medical Center, Pittsburgh, PA, United States; ^3^ Rangos Research Center, Children’s Hospital, University of Pittsburgh Medical Center, Pittsburgh, PA, United States

**Keywords:** sex, differences, GBM, gliomas, personalized

## Abstract

**Introduction:**

Glioblastoma multiforme (GBM) is one of the most aggressive types of brain cancer, and despite rigorous research, patient prognosis remains poor. The characterization of sex-specific differences in incidence and overall survival (OS) of these patients has led to an investigation of the molecular mechanisms that may underlie this dimorphism.

**Methods:**

We reviewed the published literature describing the gender specific differences in GBM Biology reported in the last ten years and summarized the available information that may point towards a patient-tailored GBM therapy.

**Results:**

Radiomics analyses have revealed that imaging parameters predict OS and treatment response of GBM patients in a sex-specific manner. Moreover, gender-based analysis of the transcriptome GBM tumors has found differential expression of various genes, potentially impacting the OS survival of patients in a sex-dependent manner. In addition to gene expression differences, the timing (subclonal or clonal) of the acquisition of common GBM-driver mutations, metabolism requirements, and immune landscape of these tumors has also been shown to be sex-specific, leading to a differential therapeutic response by sex. In male patients, transformed astrocytes are more sensitive to glutaminase 1 (GLS1) inhibition due to increased requirements for glutamine uptake. In female patients, GBM is more sensitive to anti-IL1β due to an increased population of circulating granulocytic myeloid-derived suppressor cells (gMDSC).

**Conclusion:**

Moving forward, continued elucidation of GBM sexual dimorphism will be critical in improving the OS of GBM patients by ensuring that treatment plans are structured to exploit these sex-specific, molecular vulnerabilities in GBM tumors.

## 1 Introduction

Glioblastoma (GBM) is the deadliest form of brain cancer, with patient survival estimated to be 12-15 months with treatment (1). Its incidence is 1.6 times higher in males than in females, regardless of geographical location, with primary tumors being more common in men and secondary tumors more common in women (2, 3). Available data have shown that like other normal positive predictors, such as a higher Karnofsky Performance Scale (KPS) and younger age, the female sex has been associated with increased survival of GBM patients (4, 5). Currently, the molecular basis for these differences in incidence and survival between sexes is not well defined nor completely understood.

**Figure 1:**
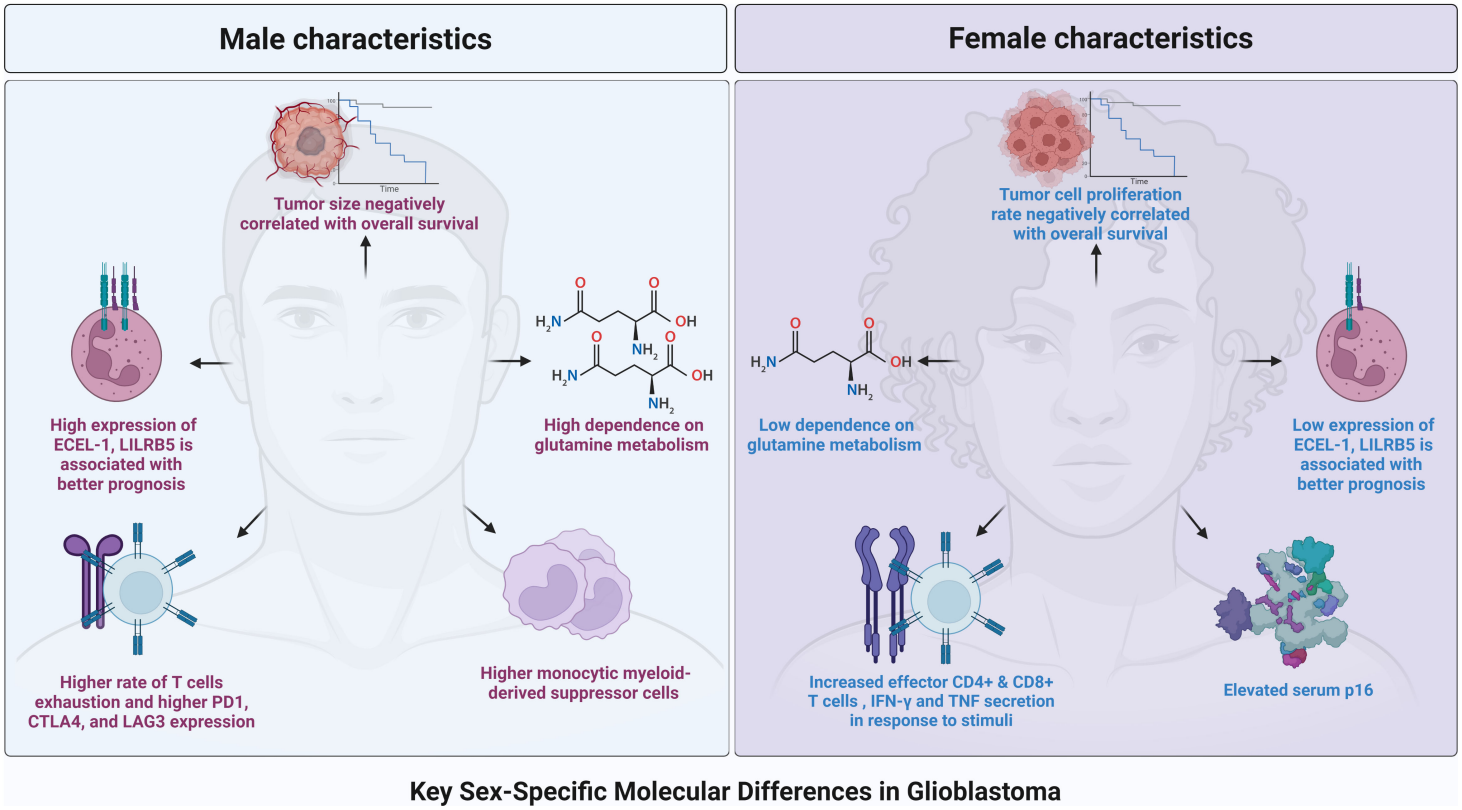
A graphical representation of the main key sex-specific difference across the landscape of glioblastoma.

While marked inter- and intra-tumoral heterogeneity is believed to be the main mechanism dictating current treatment resistance, sex-specific differences in gene expression and activity have started to gain recognition for their role in influencing the distinct pathogenesis and treatment response of GBM tumors (**Figure 1**) (5–9). It has been long established that female immune systems are more robust and responsive to foreign stimuli than male immune systems, putting patients at differential risk for malignancy and influencing tumor pathogenesis in a sex-dependent manner (10, 11). Furthermore, recent studies have revealed sex-dependent differences in tumor transcriptomes and metabolic pathways, leading to unique, sex-dependent vulnerabilities of GBM tumors to biological inhibitors and immunotherapeutics (12). These data suggest that patient sex is an important, independent factor in deciding on effective treatment regimens for GBM patients. This review aims to describe our current understanding of the sex-dependent transcriptome, metabolic, and immunological differences that affect GBM patients’ therapeutic susceptibility and overall survival.

## 2 Magnetic resonance imaging radiomics based signatures

Radiomics and radiogenomics are emerging non-invasive techniques that allow for the characterization of various imaging features that have potential prognostic value in brain tumor treatment (13). MRI imaging characteristics of FLAIR, T1, and T2, and can incorporate quantitative factors such as intensity, volume, shape, and textural variations and be contextualized with genetic data to increase the accuracy of predicted survival outcomes of patients with GBM. Several studies have shown this improved accuracy in predicting PFS and OS with the addition of a radiomics model (14–17), as well as an improved ability to predict patients’ response to treatment (18).

Thus far, only a few studies have started to elucidate gender differences in how specific imaging characteristics can be leveraged to identify OS and treatment response in GBM. In a study by Whitmire et al, MR images from over 1400 GBM patients were divided into four groups: short-term survivors (STS), non-STS, extreme survivors (EXS), and non-EXS. Using clinical and MR characteristics such as age, T1Gd radius (total tumor size), necrosis radius, CE thickness, T2/FLAIR radius, PIHNA D (tumor cell diffuse invasion rate), PIHNA ϱ (tumor cell proliferation rates), and PI D/ϱ, they found that age (HR = 1.030, p < 0.001) and T1Gd radius (HR = 1.027, p = 0.044) were significantly negatively correlated with OS in males while age (HR = 1.021, p = 0.006) and PIHNA D (HR = 1.011, p < 0.001) were significantly negatively correlated with OS in females.

Another study by Beig et al. looked at the sex-specific impact of radiomic phenotype—such as peritumoral edema, enhancing tumor, and presence of a necrotic core—on the OS and treatment response of GBM patients. In males, they identified 8 prognostic radiomic features from the radiomic phenotypes and found that capturing spots and ripple-like patterns from the enhancing tumor and peritumoral edema region were correlated with “high risk” patients (p_enhancing-tumor_ = 0.02 and p_edema_ = 8.39 × 10^−8^ respectively). In females, 6 features were obtained and showed that Laws energy features, which detect levels and edges, were associated with “low risk” patients (p_necrosis_ = 0.01 and p_edema_ = 0.0003 respectively) (19). Similarly, another study by Colen et al. found that a high volume of necrosis on tumor imaging was negatively correlated with OS in females but not males (20). These results suggest that radiomic parameters may be reflective of differences in growth features and potentially the aggressivenss of GBM tumors in a sex-specific manner.

While these radiomic models identify dimorphic patterns of radiomic parameters in GBM, more studies must be done to fully understand the sex-specific prognostic implication of these imaging features in GBM patients.

## 3 Transcriptomic landscape

Ribonucleic Acid (RNA) and protein analysis of *in vitro* and *in vivo* GBM tissue has found various autosomal genes that are differentially expressed between male and female GBM tumors. Specifically, female subjects have been found to be enriched in genes related to cell division, the G1/S transition, and the ERK1 and ERK2 cascade, while male subjects have been found to be enriched in genes related to the inflammatory response, angiogenesis, and response to tumor necrosis factor (3). High expression of the mitotic protein, epithelial cell transforming 2 (ECT2), has been linked to better survival in females, but not males (3, 21). Conversely, low expression of the immune signaling cytokine tumor necrosis factor ligand superfamily member 13B has been linked to better survival in males, but not females (3, 22). These data suggest that certain pathways may play distinct roles in the pathogenesis of GBM depending on the sex.

Multiple analyses have confirmed a relationship between differential gene expression and survival in male versus female GBM patients (8, 23). In a study by Yang et. al., five male and five female GBM gene clusters were defined by 116 common genes between the clusters, 177 genes unique to male clusters, and 167 unique to female clusters. Of these clusters, one female (fc3) and two males (mc 3 and mc5) were found to have prolonged disease-free survival (DFS) compared to the other clusters within their respective sex (8). This survival benefit was found to persist independent of IDH1 mutational status. Examination of the differentially expressed genes in the female clusters showed that the Integrin signaling pathway distinguished fc3 most significantly from the other female clusters (p<0.001), with downregulation of this pathway correlating with better survival in female patients. Similarly, the mc5 cluster was found to be associated with cell cycle pathways more significantly than the other male clusters (p<0.001), with downregulation of this pathway correlating with better survival of male patients. Although many of the downregulated cell cycle genes in mc5 were also significantly downregulated in fc3, their downregulation had a greater effect on male overall survival than female overall survival (8). In another study by Khan et. al., Kaplan-Meier analysis of differentially expressed genes between male and female GBM patients revealed that high expression of the genes ECEL-1, LILRB5, and ECEL-1 was associated with a better prognosis in males, while low expression of these genes was associated with a better prognosis in females. This incongruent effect on male and female GBM patients was also seen with the expression of other genes, such as NECAB2 (23).

This differential effect of gene expression on male and female GBM patient survival led Zhang et al. to analyze the timing of driver mutations within GBM tissue in a sex-specific manner (9). Clonal mutations derive from early tumor progenitors, and thus, are shared by most cancer cells within a patient, while subclonal mutations derive from later tumor progenitors and are only present within a subset of cancer cells (24). Integrated framework analysis revealed that mutation burden was higher in female patients, regardless of glioma grade and X chromosome status (p<0.001, FDR <0.05). Notably, females had a higher subclonal mutational burden (GBM female median = 38 vs. GBM male median = 33.5, p=0.00168) but a similar clonal mutational burden to males, unless females’ GBMs were of the classical or mesenchymal subtype (classical, p= 0.034; mesenchymal, p= 0.0017) (9). Looking at common cancer driver mutations—such as tumor protein 53 (TP53), phosphate and tenesin homolog (PTEN), and neurofibromatosis type 1 (NF1)—also revealed sex-specific clonal statuses, with mutation of these genes having a clonal tendency in mesenchymal females but a subclonal tendency in mesenchymal males (9). These results suggest that the efficacy of anti-tumor drugs that target these mutations may vary for GBM patients in a sex and subtype-dependent manner.

### 3.1 Mechanisms dictating transcriptome differences

The higher disease incidence and lower overall survival of males with GBM have led to the investigation of sex-specific differences in cell-intrinsic tumor progenitors. Various studies manipulating the expression of oncogenic drivers in both female and male astrocytes have elucidated a sex-specific difference in cell response to the activation of these oncogenic pathways (25, 26).

#### 3.1.1 Retinoblastoma protein & tumor suppressor p16

Retinoblastoma protein (Rb), a tumor suppressor that regulates the cell cycle G1/S transition, has been implicated in the tumorigenesis of many cancers, and more recently, has been connected to the sexual dimorphism seen in the aggressiveness of male versus female GBM tumors (25, 27). Given that the mesenchymal subtype of GBM occurs more frequently in males, the ability of male and female astrocytes to transform in the presence of oncogenic driver mutations has been tested (25, 28). *Nf1^-/-^* male and female astrocytes transduced with a dominant-negative p53 (DNp53) retrovirus was shown to have different growth rates, with transformed male astrocytes exhibiting a greater increase in growth as a consequence of p53 loss when compared to female astrocytes incurring the same loss (25). Implantation of neither the *Nf1–/– DNp53* male or female astrocytes was sufficient to induce tumorigenesis in mice. However, implantation of EGF-treated *Nf1–/– DNp53* male astrocytes was able to induce tumorigenesis in 100% of mice, while EGF-treated *Nf1–/– DNp53* female astrocytes were only able to induce tumorigenesis in 36% of mice (p<0.0001), suggesting that male astrocytes were more permitting to oncogenic transformation (25).

Increased oncogenic transformation in male astrocytes was accompanied by an increased percentage of these astrocytes being in the S and G_2_/M phases when compared to female astrocytes. Analysis of cell-cycle checkpoint inhibitors revealed that *Nf1–/– DNp53* male astrocytes have greater time-dependent phosphorylation of retinoblastoma protein (RB), which is a tumor suppressor, resulting in a greater level of E2F-dependent transcription and less cell cycle regulation when compared to *Nf1–/– DNp53* female astrocytes (25, 29).

In a similar model, the molecular basis of this oncogenic transformation was further investigated (30). *Nf1–/– DNp53* male and female astrocytes were subjected to serum deprivation, during which male astrocytes continued to proliferate and female astrocytes underwent almost complete growth arrest. The tumor suppressor p16, a critical inhibitor of Rb, was found to be significantly elevated in serum-deprived female, but not male, astrocytes (30, 31). Treatment of astrocytes with a cyclin-dependent kinase inhibitor (CDKi), Palbociclib, was more effective in male GBM astrocytes, suggesting that sex differences in intrinsic tumor suppressor (p16) function may underlie this sex-dependent growth arrest (30).

#### 3.1.2 Tumor protein p53

Like p16, the sex-specific activity of p53, a tumor suppressor that regulates the G1/S cell cycle transition, has been implicated to play a role in the difference in growth and volume of male versus female GBM tumors (26, 32). In a study by Rockwell et. al., the expression of *Trp53R172H*, *Trp53Y202C*, and *Trp53Y217C* was studied in male and female astrocytes. High expression of p53^R172H^ in female astrocytes and p53^Y202C^ and p53^Y217C^ in male astrocytes was able to increase growth in comparison to p53 KO astrocytes; Inoculation of these transformed astrocytes into mice led to a higher percentage of *in vivo* tumor formation and higher volume tumors when compared to astrocytes of the opposite sex with the same mutation (p53^R172H^ female 83.3% vs. male 16.7%; p53^Y202C^ female 50% vs. male 66.6%; p53^Y217C^ female 16.6% vs. male 100%) (26). This sex-dependent activity was confirmed via RNA-seq, with p53^R172H^ expression in females and p53 ^Y217C^ expression in males resulting in more differentially expressed genes than the same mutation in the opposite sex. Kyoto Encyclopedia of Genes and Genomes (KEGG) pathways, signaling cascades commonly altered in various cancers, were among the genes increasingly upregulated in these astrocytes (26, 33).

### 3.2 Methylation pattern

Analysis of The Cancer Genome Atlas for differences in methylation between male and female GBM patients has shown that various glioma subtypes have distinctive, sex-specific differentially methylated probes (DMPs) and differentially methylated regions (DMRs), and thus, that methylation patterns may also play a role in transcriptome differences between sexes (34). DMPs hyper-methylated in males consisted of cell cycle phase transition genes, while those hyper-methylated in females consisted of transcriptional regulators. These regions were associated with sex-specific binding motifs (RNA polymerase II and *E2F1* in females and *TP53* and *TCF7* in males). Importantly, *KLF6* genes in apoptotic signaling were found to be significantly downregulated in male *IDHwt* GBM patients in comparison to females, while *NFAT5* genes associated with cell migration were significantly downregulated in all female GBM patients compared to males, unveiling a possible mechanism for the sexual dimorphism of aggressivity of male versus female GBM tumors (34).

## 4 Sex-specific GBM metabolism

Differences in tumor metabolism between male and female GBM patients are being investigated (12, 35). In a study by Sponagel et. al., differential enrichment of metabolites was assessed between male and female GBM surgical specimens. Almost all of the metabolites that were differentially expressed were enriched in males (p < 0.0001), with pyroglutamine being the most enriched in comparison to females (35). Higher requirement of male GBM tissue for glutamine was confirmed via isotope labeling ([^18^F]FGln) and was independent of isocitrate dehydrogenase (IDH) status and tumor grade, with male transformed astrocytes taking up 1.5 times more the amount of glutamine as female transformed astrocytes. This dependence on glutamine correlated to male-transformed astrocytes being more sensitive to glutaminase 1 (GLS1) inhibition, while pyruvate carboxylase-mediated TCA cycle replenishment by glucose was more active in female-transformed astrocytes. Though underpowered, this study presents robust data using both human samples and *in vitro* cell work and suggests that men may exhibit response to therapeutic targeting of glutamine metabolism clinically while women may not (35). Male-transformed astrocytes have also been shown to have higher BCAT1 protein levels, making them simultaneously more susceptible to branched-chain amino acid (BCAA) deprivation (36).

In addition to differences in metabolism being implicated in sex-specific treatment response, it has also been implicated in the difference in GBM survival between sexes (12). Analysis of glycolytic gene expression in male and female GBM tissue showed that both male and female patients with high-glycolytic expression had significant differences in the mutational burden of common oncogenic drivers (EGFR, PTEN, etc.) when compared to patients with low-glycolytic expression, only males, and not females, in the high-glycolytic group had decreased OS compared to low-glycolytic patients of their respective sex (male high glycolytic median OS = 41.46 mos vs. male low glycolytic median OS = 98.16, p = 0.0005; female high glycolytic median OS = 146.02 mos vs. female low glycolytic median OS = 78.15 mos, p = 0.31113) (12). Similar sexual dimorphic effects were seen in response to lactate (lac) and pyruvate (pyr) levels, with males having elevated lac/pyr doing poorly (p = 0.0497) compared to males with a low lac/pyr, while females with an elevated lac/pyr had no significant difference in survival (p = 0.2367) compared to females with low lac/pyr (12).

## 5 Sex-specific GBM immune system characteristics

Females have been found to have a more active adaptive immune system, and thus, differential immune responses and sensitivities between sexes have been implicated in sexual dimorphism in GBM (37–39). In a study by Shireman et. al., males and females were found to have differentially composed immune systems, with males having a higher frequency of natural killer cells and females having a higher frequency of CD4^+^ T cells; This increased ratio was accompanied by enriched antigen processing and presentation (p < 0.008) and chemokine response (p < 0.008) of females in comparison to males (37). Other studies have also found an increased CD4+, and in some cases, CD8+ T cell popular in female tumors (11). Furthermore, in a recent study, male CD8+ T cells infiltrating murine GBM tumors were found to express more inhibitory receptors—such as PD1, CTLA4, and LAG3—leading to a higher rate of T cell exhaustion than in females (40). Subsequent analysis showed that while male CD8+ T cells were enriched for the stem-like/progenitor exhausted (PEX) subtype (CD8^+^CD44^+^PD1^+^TCF1^+^TIM3^-^), female CD8+ T cells were enriched for the effector (EFF) subtype (CD8^+^CD44^+^TCF1^-^TIM3^-^), and consequently, they produced more IFN- γ and TNF in response to stimuli (40). Sex differences in myeloid-derived suppressor cells (MDSCs), immature myeloid cells that can suppress the immune response and work synergistically with cancer cells, between GBM patients have also been discovered (41). In a study by Bayik et. al., monocytic myeloid-derived suppressor cells (mMDSC) were enriched in male tumors while granulocytic myeloid-derived suppressor cells (gMDSC) were enriched in female mice post-tumor implantation (38).

This dimorphism of the immune system between sexes has been shown to influence survival, with both specific gene enrichment of the adaptive immune system (TREM2, CD74, and CYTIP) and a low mMDSC/gMDSC tumor ratio and increased peripheral gMSDC expression correlating to a significantly increased OS in female murine GBM models compared to male murine GBM models (37, 38). Moreover, this differential immune genetic profile influences the efficacy of therapeutics in the female and male GBM population. Anti-PD1 monoclonal antibody (mAb) treatment has been shown to greatly increase the survival of male GBM murine models but not female GBM murine models, expectedly due to the greater frequency of T cell exhaustion markers in the male T cell tumor population (11). Similarly, therapeutics targeting mMSDCs (fludarabine) versus gMDSCs (anti-IL1β) had sex-dependent effects on GBM tumor growth in murine models, with fludarabine treatment decreasing tumor growth solely in males and anti-IL1β treatment decreasing tumor growth solely in females (38).

Meta-analysis of GBM immunotherapy clinical trials’ data has confirmed that the efficacy of immunological therapy is sex-dependent, with the OS of female patients receiving immunotherapy at 1 year being significantly higher than male patients (p = 0.0241). Even better OS was seen when the immunotherapy (autologous dendritic cells) was tailored to the immune landscape of female patients (p = 0.0158) (37). Cumulatively, these results suggest that the efficacy of immunotherapy is sex-dependent, and tailoring patients’ care regimens to exploit the sex-specific differences in the immune compartment could improve GBM patient OS and progression-free survival (PFS).

## 6 Conclusion and future direction

The complex effect of sex on the pathogenesis and survival of GBM has begun to be elucidated but is still not completely understood. Radiomics analysis of GBM tumors has revealed sexual dimorphism in the prognostic implication of various imaging features. Additionally, genetic analysis of male and female tumor specimens has revealed a differential expression of cell division, inflammatory signaling, and angiogenesis genes between tumor tissue of the two sexes (3, 21).

Standard molecular profiling of GBM tumors, as well as sex-specific treatment regimens, are necessary to overcome the stagnation that has been seen in GBM survival over the last few decades. Molecular sex-dependent vulnerabilities and sex-dependent resistances elucidated in the aforementioned studies should be taken into consideration when building male and female patient treatment plans. Screening for these sex-specific oncogenic drivers and optimizing the targeting of these sex-specific, molecular vul5 nerabilities could allow for implementation of treatments that are more cytotoxic to GBM tumors on a cellular level. Such personalized therapy has the potential to improve the overall survival of these patients. For instance, increased 1-year OS of female patients after immunotherapy suggests that these drugs may be better suited for females and that males may require tailored immunotherapies to support an already lacking immune response (37, 42). As molecular techniques continue to advance and our understanding of the pathogenesis of these tumors increases, it is important that we subsequently change our perception of the “standard of care” in order to understand and treat the heterogeneity of GBM through personalized therapies.

## Author contributions

NJ: Writing – original draft, Writing – review & editing. AH: Resources, Visualization, Writing – original draft, Writing – review & editing. AC: Writing – original draft, Writing – review & editing. NH: Writing – review & editing. HD: Writing – review & editing. RS: Writing – review & editing. JH: Writing – review & editing. PZ: Conceptualization, Supervision, Validation, Writing – original draft, Writing – review & editing.
